# Bone‐wise rigid registration of femur, tibia, and fibula for the tracking of temporal changes

**DOI:** 10.1002/acm2.70053

**Published:** 2025-03-11

**Authors:** Arttu Ruohola, Ville Haapamäki, Eero Salli, Tuomas Kaseva, Marko Kangasniemi, Sauli Savolainen

**Affiliations:** ^1^ HUS Diagnostic Center Department of Radiology University of Helsinki and Helsinki University Hospital Helsinki Finland; ^2^ Department of Physics University of Helsinki Helsinki Finland

**Keywords:** bone structures, convolutional neural network, CT, multiple myeloma, rigid registration

## Abstract

**Background:**

Multiple myeloma (MM) induces temporal alterations in bone structure, such as osteolytic bone lesions, which are challenging to identify through manual image interpretation. The large variation in radiologists' assessments, even at expert centers, further complicates diagnosis. Automatic image analysis methods, including segmentation and registration, can expedite detecting and tracking these bone changes.

**Purpose:**

This study presents an automated pipeline for accurately tracking temporal changes in the femurs, tibiae, and fibulae of MM patients using 3D whole‐body CT images. The pipeline leverages image segmentation, rigid registration, and temporal subtraction to accelerate disease monitoring and support clinical decision‐making.

**Methods:**

A convolutional neural network (CNN) was trained to segment bones in 3D CT images of 30 MM patients. Nine patients with pre‐ and post‐diagnosis CT scans were used to validate the segmentation and registration process. A two‐phase bone‐wise rigid registration was applied, followed by temporal subtraction to generate difference images. Segmentation and registration accuracy were assessed using the Dice similarity coefficient (DSC) and mean surface distance (MSD). The proposed method was compared to a non‐rigid registration method.

**Results:**

The neural network segmentation resulted in a mean DSC of 0.93 across all bone types and all test data. The registration accuracy measured by the mean DSC across the test data was at least 0.94 for all bone types. The second phase of rigid registration improved the registration fibulae. Metric‐wise, the nonrigid method performed better but diminished lesion visibility in difference images.

**Conclusions:**

An automated pipeline for the longitudinal tracking of bone alterations was presented. Both segmentation and registration demonstrated high accuracy as measured by DSC and MSD. In the difference images produced by temporal subtraction, osteolytic lesions were clearly visible in the femurs. The methodology lays a solid foundation for future improvements, such as inclusion of the axial spine.

## 1 INTRODUCTION

Specific diseases, including multiple myeloma (MM), often present symptoms in the form of temporal alterations in bone structure, such as the development of osteolytic bone lesions in the case of MM. In short, MM is a complex disease that evolves from asymptomatic premalignant stages and progresses to symptomatic MM. One of the major difficulties in MM is that the disease definition is mostly clinicopathological, and it needs explicit clinical manifestations of end‐organ damage, such as osteolytic bone lesions and renal failure, before the diagnosis can be made.^[^ ^1^ ^]^ Bone imaging in MM is important for diagnosis since osteolytic lesions in radiographs are a reason for treatment. Myeloma bone destruction also represents a major cause of morbidity and delay in the diagnosis of MM, and postponement of therapy could be harmful to these patients. One weakness of radiographic detection is that it may reveal osteolytic disease only when over 30% of the trabecular bone has been lost.^[^ ^2^ ^]^. In the past 20 years, a low‐dose whole‐body computed tomography (LDCT) has been widely used to assess bone disease and bone marrow infiltration in MM.^[^ ^3^ ^]^ However, small bone lesions can pose a challenge to human perception, and care must also be taken not to interpret insignificant benign lytic bone changes as pathological on LDCT. If there are doubts about the nature of these lesions, a repeat LDCT scan in 3–6 months is usually done before a diagnosis of MM is made.

Identifying bone alterations and lesions by manually reviewing numerous slices of 3D medical images is a time‐consuming and tedious task for radiologists. Also, the large variation between readers in the interpretation of images, even in university‐level centers, is a common challenge.^[^ ^4^ ^]^

Significant expeditions in the efficiency of temporal tracking for bone alterations can be achieved by leveraging a spectrum of automatic image analysis operations and methodologies, such as image segmentation and registration.^[^ ^5^ ^]^ An effective computer‐aided detection (CADe) strategy for automating the tracking of temporal alterations involves segmenting and registering a structure of interest from two medical images obtained at distinct time points. Subsequently, temporal subtraction (TS) is performed. In TS, two registered medical images obtained at distinct time points are subtracted to generate a difference image. Ideally, a difference image contains only information pertaining to the alterations that occurred between the two time points. A difference image generated in this fashion could then be overlaid on the latest medical image available to highlight regions of temporal change in diseases, such as MM.

Various applications of image registration and TS in longitudinal bone tracking are known in scientific literature. However, commonly these applications have utilized nonrigid registration^[^ ^6^, ^7^, ^8^, ^9^, ^10^, ^11^, ^12^ ^]^ and traditional algorithmic segmentation.^[^ ^13^ ^]^ The use of nonrigid registration can be thought to reduce temporal subtraction errors^[^ ^7^, ^14^, ^15^ ^]^; however, such methods may also produce false fitting. Applying nonrigid transformations to bones, that are rigid objects, may result in the loss of temporal changes in TS or generate false changes. The use of rigid bone‐wise segmentation and registration prior to TS circumvents this issue, possibly resulting in more valid registrations.

Some authors report the use of rigid registration in TS. Kemp et al.^[^ ^16^ ^]^ demonstrated success in detecting longitudinal microarchitectural changes in the vitamin D participant cohort of the Calgary Vitamin D Study^[^ ^17^ ^]^ by rigidly registering high‐resolution peripheral quantitative CT images. Horger et al.^[^ ^18^ ^]^ used automatic segmentation and rigid registration to calculate CT‐TS for MM patients' axial spine. However, Horger et al.^[^ ^18^ ^]^ reported using a prototype of commercial software (bone subtraction, Syngo.via CT software package, Siemens Healthcare), and the analysis does not appear to be reproducible with the given descriptions. Kemp et al.^[^ ^16^ ^]^ also reported the use of commercial registration software associated with a particular scanner, making their analysis unrepeatable without the purchase of expensive hardware and software that may or may not be available anymore.

In this study, we introduce an automated and reproducible pipeline incorporating publicly available, state‐of‐the‐art segmentation, and registration methodologies. We present an entirely reproducible and open‐source software‐based approach, in which we introduce convolutional neural network (CNN) segmentation, combined with rigid bone‐wise registration, for the computation of CT‐TS measurements across multiple bones in patients afflicted with MM. This pipeline is designed to aid accurate tracking of temporal changes within the femurs, tibiae, and fibulae—although the tool could easily be expanded to include most of the bones. We utilized a CNN for 3D image segmentation, followed by a two‐phase bone‐wise rigid registration process and subsequent difference image calculation. This methodology was applied to 3D whole‐body CT images of MM patients, for which two distinct time point images were available. We also compare our method to a simple baseline rigid registration and to a nonrigid registration method.

This integration of CNN segmentation with rigid registration facilitates the determination of TS values for any object of interest without necessitating algorithmic modifications. Our research aims to provide a robust, reproducible, and expandable methodology for CT‐TS assessment, which is essential for advancing the field of MM diagnosis and treatment.

## 2 MATERIALS AND METHODS

### 2.1 Subjects

The ethical committee of Helsinki University Hospital approved this retrospective study (HUS/211/2020), and informed consent was waived. The data for this study were retrieved from the Siemens Picture Archiving and Communications System (syngo.share webview diagnostic VA31C, Siemens Healthineers, Germany), which facilitated the reassessment of whole‐body LDCT scans conducted at Helsinki University Hospital, Helsinki, Finland, between April 2016 and November 2022. The initial dataset comprised 203 patients diagnosed with MM. From this pool, 39 patients were selected based on the inclusion criterion of having documented MM‐induced lesions in their femurs. Of these 39 patients, 30 had only post‐onset 3D CT images available. These 30 patients were employed to train and validate the CNN for bone segmentation, with 26 patients used for training and four reserved for model validation. Nine patients had paired 3D CT images that included both pre‐MM (before the clinical onset of MM) and post‐MM (after the onset) scans. The nine paired CT images were utilized to test the complete segmentation and registration pipeline presented in this study. These nine patients consisted of six males and three females, aged between 64 and 84 years, with a mean age of 75.

### 2.2 Image acquisition

Whole‐body 3D CT images were acquired using four scanners, SOMATOM Definition Flash (Siemens Healthineers, Erlangen, Germany), SOMATOM Force (Siemens Healthineers, Erlangen, Germany), SOMATOM Force (Siemens Healthineers, Erlangen, Germany), and LightSpeed VCT (GE Healthcare, Chicago, USA). The images acquired using SOMATOM Definition Edge, SOMATOM Definition Flash, and LightSpeed VCT were acquired using 120/35 kV/mA and SOMATOM Force was operated at 100/75 kV/mA or 100/74 kV/mA. The data used in this work was acquired from patient data archives retrospectively, thus varying scanner models, tube voltages, and amperages are present in the data set. Imaging parameters of the patients used in the training and validation of the bone segmentation neural network were: SOMATOM Definition Edge (125 kV, 35 mA, *N* = 26) and SOMATOM Force (100 kV, 75 mA, *N* = 4). The 200 most superior slices were cropped out of all images. The voxel spacing of the images varied between from 0.855 to 0.976 mm, from 0.855 to 0.976 mm, and from 2.99 to 3.00 mm in *x*‐, *y*‐ and *z*‐directions, respectively.

### 2.3 Manually corrected algorithmic bone segmentations

**FIGURE 1:**
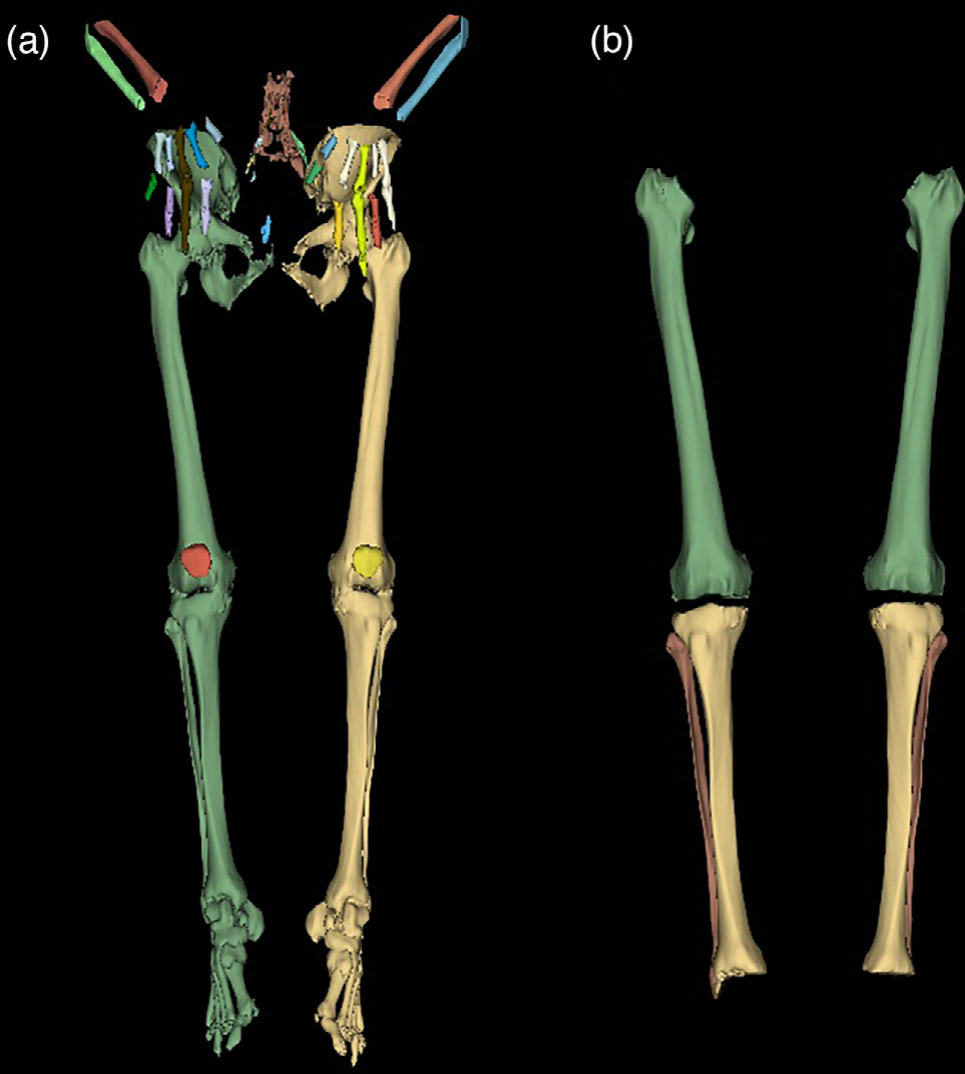
Results of the algorithmic bone segmentation and the applied manual corrections. (a) Output of the bone segmentation algorithm by Krcah et al.^[^ ^19^ ^]^ Note that femur, tibia, fibula, pelvic bones and the bones of the metatarsus are all fused. (b) The bones of interest (femurs, tibiae and fibulae) have been manually separated from the fused segmentation using 3DSlicer's 3D cutting tool, and each type of bone has been given a unique label.

The initial segmentation of femur, tibia, and fibula bone structures was achieved through automated processing of whole‐body 3D CT images using the methodology outlined by Krcah et al.^[^ ^19^ ^]^ This approach employs an energy minimization segmentation strategy rooted in combinatorial graph theory, coupled with a bone boundary enhancement filter that analyzes second‐order local structures.^[^ ^19^ ^]^ The default parameters, as delineated by Krcah et al.,^[^ ^19^ ^]^ were utilized for the bone segmentation pipeline. Subsequently, the initial label maps generated by this segmentation pipeline underwent manual refinement to separate the bones from each other by using the 3DSlicer software^[^ ^20^ ^]^ to eliminate the need for iterative parameter fine‐tuning. Figure 1a shows the output of the algorithmic bone segmentation and Figure 1b shows the outcome of the manual refinements. In the described manner, 30 3D CT images were segmented for training and validating a CNN for bone segmentation and the nine pre‐ and post‐MM images to serve as ground truth for testing the pipeline. The ground truth segmentations were inspected and approved by a radiologist.

### 2.4 Neural network bone segmentation

For the segmentation of femurs, tibiae and fibulae, we employed the VNet neural network,^[^ ^21^ ^]^ edited to allow a flexible number of filters. The training of this network was carried out using the MONAI framework,^[^ ^22^ ^]^ an open‐source project built on PyTorch,^[^ ^23^ ^]^ emphasizing best practices in deep learning for medical imaging applications.

**FIGURE A1:**
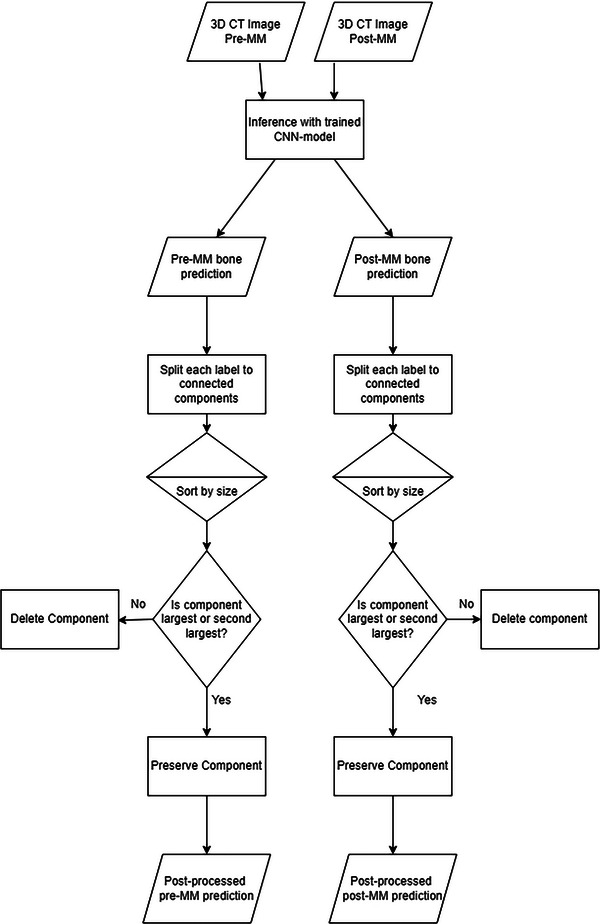
A flowchart depicting the CNN segmentation process and prediction post‐processing. CNN, convolution neural network.

Prior to inputting the data into the neural network, we performed intensity scaling, transforming intensity values from the range [‐500, 3000] to [0, 1]. Subsequently, we applied foreground cropping to enhance learning and computational efficiency. The dataset was then subjected to random cropping, incorporating both positive and negative samples, ensuring the acquisition of valuable training data for the model. To augment the dataset further, a rotational transformation was applied to the first two spatial dimensions of the data with a probability of p = 0.50, within the range of [$\frac{-\pi }{4}$, $\frac{\pi }{4}$] radians. During processing, the data was divided into 320 × 320 × 64 patches before being fed into the neural network. The model was trained for 2000 epochs and validated every 50 epochs. The batch size was set to one. The size of the training set was 26 images and the validation set was four images. The Adam optimizer was used with a learning rate of 0.001. As the loss function we employed the mean of Dice score and cross‐entropy loss. The dropout probability of the last layer of the CNN was set to 0.3. Segmentation accuracy was evaluated by calculating Dice similarity coefficients (DSCs) and mean surface distances (MSDs) for the validation and test data sets, using the manually corrected algorithmic segmentations as ground truths. Before calculating the performance statistics, the predictions were post‐processed by including only the two largest components per label. Figure A1 in the Appendix visualizes the segmentation process.

**FIGURE 2:**
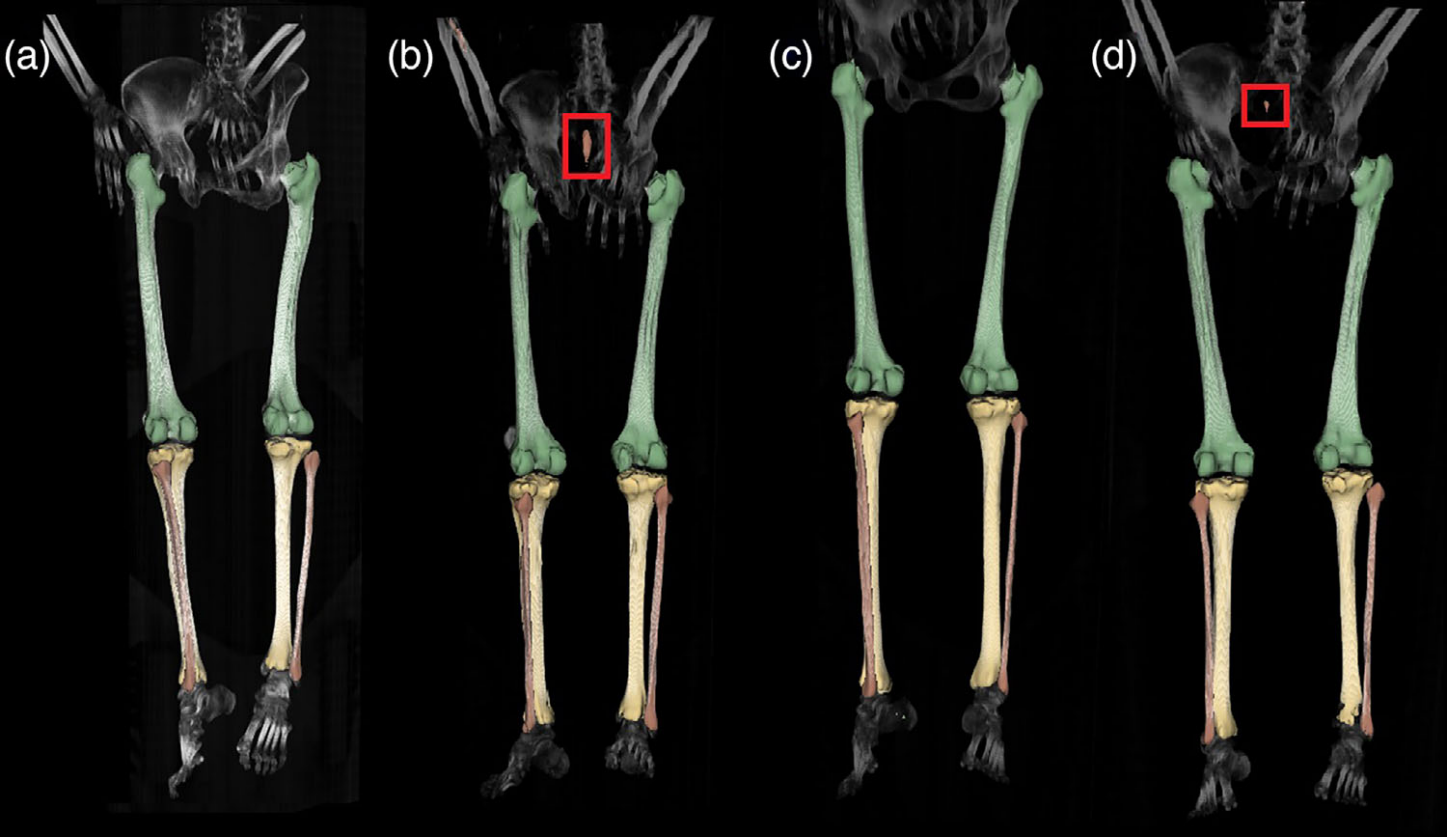
Predictions of the bone segmentation CNN. (a)–(d) Predictions of the CNN over the validation dataset. The predictions are overlaid on maximum intensity projection images. Red squares highlight false positive predictions where the CNN incorrectly predicted bone structures or included additional regions. CNN, convolutional neural network.

**FIGURE 3:**
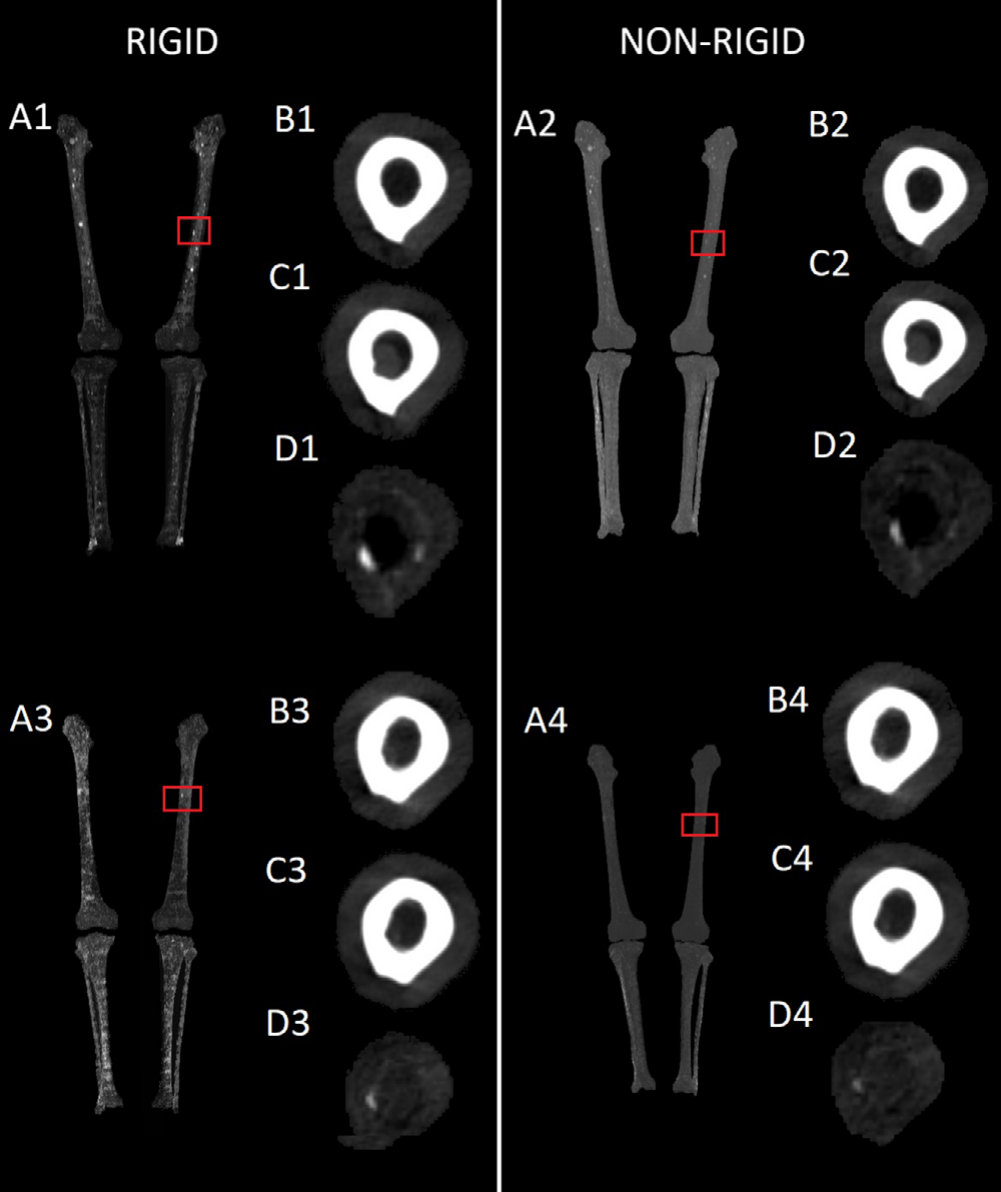
Coronal slice of registered segmentation masks in radiological convention with the overlaid contour lines of the fixed images. A blue contours are used for the fixed images and red masks for the moving images. (a) Femurs. (b) Tibiae. (c) Fibulae.

**FIGURE 4:**
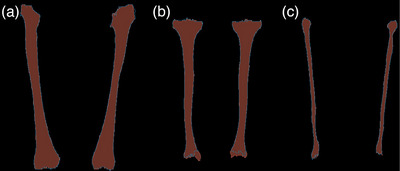
(a1–a4) Maximum intensity projections of difference images of the six bones in radiological convention. The red squares highlight osteolytic bone lesions visible in the difference images. (b1–b4) Axial slices of the pre‐MM images. (c1–c4) Same axial slices of the post‐MM images. In c1 bone marrow infiltrate can be seen in the center of the bone as a gray mass. (d1–d4) The same axial slices of the difference images, generated by temporal subtraction. The osteolytic lesions are visible as voxels of higher intensity. Marrow infiltrate is visible in black. MM, multiple myeloma.

**FIGURE 5:**
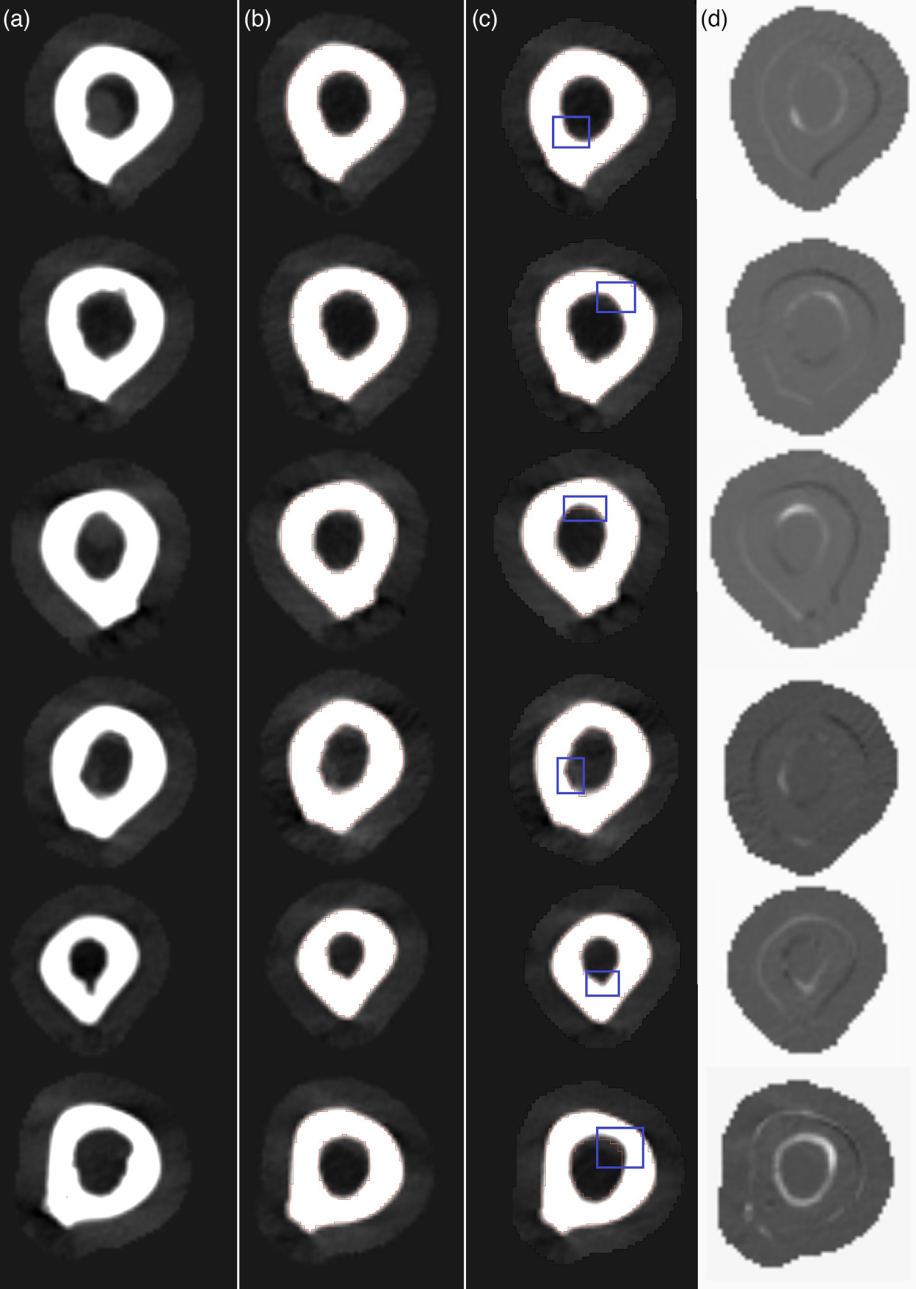
Column (a) Slices of post‐onset MM images. Column (b) The same slices in rigidly registered pre‐onset images. Column (c) The same slices in nonrigidly registered pre‐onset images. The blue squares highlight an area where differences can be seen between rigid and nonrigid methods. Column (d) Difference images of rigidly registered pre‐onset images and nonrigidly registered pre‐onset images show that the nonrigid method creates a lesion‐like dent into the moving pre‐MM images. MM, multiple myeloma

### 2.5 Rigid bone registration

To ensure consistently high levels of accuracy in registrations, without manual intervention, we implemented a two‐stage bone‐wise registration pipeline. Each bone was subjected to the registration pipeline individually. This pipeline entails an initial multiresolution rigid registration, succeeded by a bounding box cropping of the specific bone under registration, and culminating in another multiresolution rigid registration process. In the initial multiresolution rigid registration, we used SimpleITK's^[^ ^24^, ^25^, ^26^ ^]^ Powell optimization with Brent line search using the parameters of Table 1. In the first registration, the entire image volume was used, but all other structures outside the CNN‐predicted bone segmentation mask were set to zero. However, each axial slice of the segmentation mask was separately two‐dimensionally dilated by using a circular kernel^[^ ^27^ ^]^ with a radius of eight voxels. The points used to calculate metric values during the registration were sampled from the dilated segmentation masks.

**TABLE 1:** Parameters of the initial registration.

Parameter	Option
Software	SimpleITK
Sampling	Regular
Metric	Mean squares
Optimizer	SetOptimizerAsPowell()
Transformation	VersorRigid3DTransform()
Number of iterations	5000
Maximum line iterations	500
Step length	1.0
Step tolerance	1e‐8
Value tolerance	1e‐8
Parameter weighting	SetOptimizerScalesFromPhysicalShift()
Shrink factors per level	[4, 2, 2, 1]
Smoothing sigmas per level	[4, 2, 2, 1]
Sampling percentage per level	[0.25, 0.50, 0.75, 1.00]
Interpolator	Lanczos Windowed Sinc
Cropping	No

**FIGURE A2:**
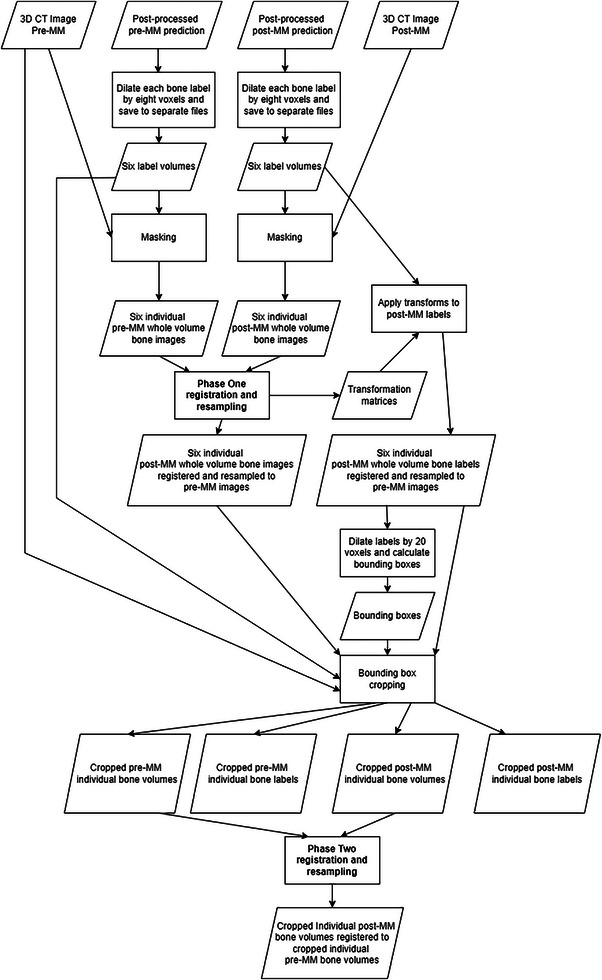
A flowchart depicting the registration process.

Following the initial registration, the segmentation mask of the bone under registration was dilated by using a circular kernel with a radius of 20 voxels to expand its boundaries. Subsequently, a bounding box was generated based on this dilated segmentation mask. This bounding box was then employed to isolate and crop the bone prior to subjecting the bone to the final registration process. In the final registration process, we used the Advanced Normalization Tools software^[^ ^28^ ^]^ configured for rigid registration with the parameters of Table 2. The post‐MM images were used as moving images. For reference, we also evaluated the registration method without the second registration phase of the pipeline and also compared our method to a simple baseline method of which parameters are displayed in Table 3. In the transformation of binary masks, nearest neighbor interpolation was used. For the baseline method and the initial registration, the label maps of the bones that were dilated by eight voxels were used as metric masks. The baseline registration was executed without cropping similarly to the initial registration. After the initial and final registration, the images were resampled with Lanczos Windowed Sinc interpolator and after the baseline registration linear resampling was used. Figure A2 in the Appendix visualizes the registration pipeline.

**TABLE 2:** Parameters of the final registration.

Parameter	Option
Software	ANTs
Sampling	Regular
Sampling percentage	0.35
Metric	Mean squares
Transformation	Rigid
Rotation	Quaternion
Maximum step size	0.1
Convergence threshold	1e‐6
Shrink factors per level	[8, 4, 2, 1]
Smoothing sigmas per level	[3, 2, 1, 0]
Max iterations per level	[1000, 500, 250, 100]
Interpolator	Lanczos Windowed Sinc
Cropping	Yes

**TABLE 3:** Parameters of the simple baseline reference registration.

Parameter	Option
Software	SimpleITK
Sampling	Random
Sampling percentage	0.1
Metric	Mean squares
Optimizer	SetOptimizerAsGradientDescent()
Transformation	Euler3DTransform()
Number of iterations	1500
Learning rate	0.1
convergenceMinimumValue	1e‐6
convergenceWindowSize	10
Parameter weighting	SetOptimizerScalesFromPhysicalShift()
Interpolator	Linear
Cropping	No

### 2.6 Nonrigid reference bone registration

To evaluate our rigid registration method in comparison with a nonrigid approach, we isolated the left and right legs of each patient by cropping them into separate volumes. These volumes were then nonrigidly registered using the symmetric normalization “antsRegistrationSyN[s]” pipeline of AntsPy^[^ ^29^, ^30^ ^]^ with default parameters. The pre‐onset images were used as moving images. For assessing temporal changes, we utilized TS, as outlined in Section 2.7.

### 2.7 Difference images

To identify temporal alterations within the registered bone structures and visually assess the registration's precision, we computed difference images by subtracting the post‐MM image from the pre‐MM image. Difference images generated in this manner show reduced density in bright and increased density in dark. We also generated differences images by subtracting nonrigidly registered images from rigidly registered images to study the differences between the two methods.

### 2.8 Evaluation metrics

To assess the accuracy of our segmentation and registration procedures, we conducted an evaluation using two well‐established metrics: the DSC^[^ ^31^ ^]^ and the MSD.^[^ ^32^, ^33^ ^]^ DSC can be calculated by

(1) $$DSC=\frac{2*|X\cap Y|}{\left|X|+|Y\right|}$$

where X and Y are the binary masks of the registered or segmented bones being compared. Larger values of DSC indicate a greater degree of similarity. Binary masks were used to calculate this metric.

The MSD is a metric used to quantify the average variation in surface position between two sets of data, typically between a registered image and a fixed image or between a segmentation result and a ground truth reference. It provides a measure, typically expressed in millimeters (mm), that indicates how much, on average, the surfaces of the two data sets differ. Smaller values of mean SD indicate a greater degree of similarity. Mathematically mean SD can be expressed as follows:

(2) $$MSD=\frac{1}{n_{S}+n_{S}^{\prime }}\left(\sum_{p=1}^{n_{S}}d\left(p,S^{\prime }\right)+\sum_{p^{\prime }=1}^{n_{S}^{\prime }}d\left(p^{\prime },S\right)\right),$$

where $n_{S}$ is the number of points in surface S, $n_{S}^{\prime }$ is the number of points in surface $S^{\prime }$, p and $p^{\prime }$ are indices running from 1 to $n_{S}$ and $n_{S}^{\prime }$ and represents individual points on the surfaces S and $S^{\prime }$, $d(p,S^{\prime })$ is the euclidean distance between point p on surface S and the nearest point on the surface $S^{\prime }$, and finally $d(p^{\prime },S)$ is the euclidean distance between point $p^{\prime }$ on surface $S^{\prime }$ and the nearest point on the surface S. Registered binary masks were used in the calculations.

In addition to DSC and MSD, registration results were further evaluated by calculating mean squared error (MSE) by

(3) $$MSE=\frac{1}{n}\sum_{i=1}^{n}{\left(y_{i}-p_{i}\right)}^{2}$$

where $y_{i}$ is the ith value in the fixed image, and $p_{i}$ is the corresponding value in the registered image. In the context of CT, the calculation of MSE will yield the unit (${\mathrm{HU}}^{2}$), where HU refers to the Hounsfield unit.

## 3 RESULTS

### 3.1 Neural network bone segmentation

To evaluate the accuracy of CNN segmentation, DSC and MSD were calculated for each bone type (femur, tibia, and fibula) for the validation group (N = 4) and for the pre‐ and post‐MM test groups (N = 9). Left and right side bones are handled as a single object in calculating the DSC and MSD for the segmentations. The results of these calculations are collected in Tables 4, 5, 6. In the validation group mean DSCs of femurs, tibiae, and fibulae across all patients were 0.98 ± 0.01, 0.94 ± 0.03, and 0.94 ± 0.03, respectively.

**TABLE 4:** DSCs and mean surface distances calculated for each bone type (femur, tibia, fibula) in the validation dataset (N = 4).

Femurs			Tibiae		Fibulae	
Patient	DSC	MSD (mm)	DSC	MSD (mm)	DSC	MSD (mm)
1	0.98	0.14	0.93	0.63	0.95	0.14
2	0.96	0.53	0.91	0.95	0.90	0.36
3	0.98	0.15	0.97	0.22	0.96	0.16
4	0.98	0.28	0.96	0.33	0.96	0.15

*Note*: The metrics evaluate the accuracy of the CNN segmentation model for identifying bone structures in 3D CT images, with higher DSC and lower MSD values indicating better performance.

Abbreviations: CNN, convolutional neural network; CT, computed tomography; DSC, Dice similarity coefficient; MSD, mean surface distance.

**TABLE 5:** DSCs and MSDs calculated over the pre‐MM test set.

Femurs			Tibiae		Fibulae	
Patient	DSC	MSD (mm)	DSC	MSD (mm)	DSC	MSD (mm)
1	0.98	0.16	0.96	0.36	0.95	0.17
2	0.96	0.50	0.96	0.38	0.87	0.27
3	0.98	0.18	0.96	0.30	0.93	0.15
4	0.99	0.15	0.94	0.51	0.91	0.27
5	0.98	0.22	0.95	0.46	0.80	0.25
6	0.98	0.26	0.92	0.75	0.85	0.28
7	0.98	0.34	0.96	0.34	0.91	0.34
8	0.98	0.27	0.95	0.47	0.88	0.21
9	0.95	0.64	0.94	0.54	0.84	0.35

Abbreviations: DSC, Dice similarity coefficient; MM, multiple myeloma; MSD, mean surface distance.

**TABLE 6:** DSCs and MSDs calculated over the post‐MM test set.

Femurs			Tibiae		Fibulae	
Patient	DSC	MSD (mm)	DSC	MSD (mm)	DSC	MSD (mm)
1	0.98	0.15	0.94	0.48	0.94	0.21
2	0.88	1.63	0.90	1.07	0.81	0.63
3	0.98	0.20	0.94	0.54	0.89	0.30
4	0.99	0.14	0.94	0.53	0.90	0.26
5	0.98	0.26	0.94	0.54	0.89	0.24
6	0.96	0.38	0.95	0.50	0.91	0.28
7	0.98	0.26	0.95	0.45	0.90	0.32
8	0.98	0.24	0.97	0.27	0.91	0.23
9	0.97	0.39	0.95	0.44	0.75	0.28

Abbreviations: DSC, Dice similarity coefficient; MM, multiple myeloma; MSD, mean surface distance.

In the pre‐MM data set mean DSCs of femurs, tibiae, and fibulae across all patients were 0.98 ± 0.01, 0.95 ± 0.01, and 0.88 ± 0.05, respectively. In the post‐MM data set the mean DSCs of femurs, tibiae, and fibulae across all patients were 0.97 ± 0.04, 0.94 ± 0.02, and 0.88 ± 0.06. The mean DSC across all bone types and all test data was 0.93 ± 0.06. The mean MSD across all bone types and all test data was 0.41 ± 0.31 mm.

The CNN predictions overlaid on maximum projection images are visualized in Figure 2. Red squares highlight false positive predictions made by the neural network.

### 3.2 Bone‐wise rigid registration

The registration pipeline was applied individually to the femurs, tibiae, and fibulae. To quantify registration accuracy, we computed the means and standard deviations for MSDs, DSCs, and MSEs for each of the six bones across the nine patients. For reference, we also calculated the evaluation metrics for registration without the second phase (one‐phase) and also with a simple baseline registration method (see Table 3). All the results are displayed in Table 7. Figure 3 visualizes the success of the registration two‐stage pipeline by overlaying the contours of a coronal slice from an example fixed (pre‐MM) and moving image (post‐MM). Red contour is used for moving images and green for fixed images. The second phase of the registration pipeline improved the registration accuracies, measured by DSC and MSD, of right fibulae (mean DSC 0.90 ± 0.11 vs. 0.93 ± 0.02 and mean MSD 0.42 ± 0.57 mm vs. 0.23 ± 0.08 mm). The MSE of tibiae and fibulae is lower after the second registration phase, indicating improvement. The baseline reference registration yielded notably worse results in all metrics (mean DSC = 0.85, mean MSD = 1.16 mm, mean MSE = 6.63 $\times 10^{3}\;{\mathrm{HU}}^{2}$) in comparison to one‐phase registration (mean DSC = 0.95, mean MSD = 0.34 mm, mean MSE = 1.33 $\times 10^{3}$ ${\mathrm{HU}}^{2}$) and two‐phase registration (mean DSC = 0.95, mean MSD = 0.31 mm, mean MSE = 1.12 $\times 10^{3}$ ${\mathrm{HU}}^{2}$).

**TABLE 7:** Bone‐wise means of DSC, MSD, and MSE (± standard deviation) across all nine patients using the simple baseline registration, one‐phase registration and two‐phase registration.

Baseline				One‐phase			Two‐phase		
Bone	DSC	MSD (mm)	MSE ($10^{3}{\mathrm{HU}}^{2}$)	DSC	MSD (mm)	MSE ($10^{3}{\mathrm{HU}}^{2}$)	DSC	MSD (mm)	MSE ($10^{3}{\mathrm{HU}}^{2}$)
Femur (L)	0.90 ± 0.05	1.03 ± 0.57	6.34 ± 5.78	0.97 ± 0.02	0.32 ± 0.23	0.54 ± 0.45	0.97 ± 0.02	0.32 ± 0.23	0.54 ± 0.45
Femur (R)	0.91 ± 0.06	0.90 ± 0.69	4.79 ± 5.37	0.96 ± 0.02	0.35 ± 0.32	0.59 ± 0.55	0.96 ± 0.02	0.35 ± 0.32	0.59 ± 0.55
Tibia (L)	0.93 ± 0.04	0.59 ± 0.40	5.55 ± 6.14	0.96 ± 0.01	0.33 ± 0.10	1.93 ± 1.16	0.96 ± 0.01	0.34 ± 0.10	1.89 ± 1.14
Tibia (R)	0.91 ± 0.06	0.85 ± 0.65	9.18 ± 11.60	0.95 ± 0.02	0.40 ± 0.15	1.91 ± 0.89	0.95 ± 0.02	0.40 ± 0.15	1.87 ± 0.84
Fibula (L)	0.69 ± 0.30	2.53 ± 4.40	8.49 ± 9.96	0.90 ± 0.11	0.42 ± 0.57	2.05 ± 3.64	0.93 ± 0.02	0.23 ± 0.08	0.91 ± 0.48
Fibula (R)	0.79 ± 0.24	1.06 ± 1.51	5.42 ± 7.75	0.94 ± 0.01	0.22 ± 0.05	0.95 ± 0.49	0.94 ± 0.01	0.22 ± 0.05	0.89 ± 0.45

*Note*: (L) and (R) refers to the patient's left and right side bones, respectively.

Abbreviations: DSC, Dice similarity coefficient; MSD, mean surface distance; MSE, mean squared error.

### 3.3 Nonrigid reference registration

The nonrigid registration method was applied leg‐wise using the pre‐MM images as moving images. The evaluation parameters DSC and MSE were calculated bone‐wise using the transformed segmentations but MSE was calculated leg‐wise. The means with standard deviations of these calculations are collected in Tables 8 and 9. The mean DSC across all bone types is 0.96 ± 0.01 and the mean MSD across all bone types is 0.24 ± 0.1. The DSC and MSD metrics of nonrigid registration are equal to or slightly better than our proposed pipeline's. Using our rigid method, MSE averages of the bones of the left leg sum to 3.34 $\times 10^{3}{\mathrm{HU}}^{2}$, and using the nonrigid method, left leg MSE average of 2.53 $\times 10^{3}{\mathrm{HU}}^{2}$ was obtained. For the right leg, the results are 3.35 $\times 10^{3}{\mathrm{HU}}^{2}$ (rigid) versus 2.53 $\times 10^{3}{\mathrm{HU}}^{2}$ (nonrigid).

**TABLE 8:** Bone‐wise mean DSCs and MSDs with standard deviations across all nine patients using the reference nonrigid registration method.

Bone	DSC	MSD (mm)
Femur (L)	0.97 ± 0.02	0.28 ± 0.23
Femur (R)	0.97 ± 0.02	0.32 ± 0.31
Tibia (L)	0.97 ± 0.01	0.18 ± 0.12
Tibia (R)	0.96 ± 0.01	0.35 ± 0.14
Fibula (L)	0.95 ± 0.02	0.18 ± 0.08
Fibula (R)	0.96 ± 0.01	0.16 ± 0.04

Abbreviations: DSC, Dice similarity coefficient; MSD, mean surface distance.

**TABLE 9:** Leg‐wise means of MSE (± standard deviation) using the reference nonrigid registration method.

Leg	MSE ($10^{3}{\mathrm{HU}}^{2}$)
Right leg	2.72 ± 1.70
Left leg	2.53 ± 1.08

Abbreviations: MSE, mean squared error.

### 3.4 Difference images

We generated two types of difference images (as described in Section 2.7) for the nine patients with pre‐ and post‐MM images available, employing the rigid pipeline and a nonrigid reference method.

Figure 4 showcases the results of TS for two patients. Figure 4 A1 and A3 present the maximum intensity projections of difference images of two patients' femurs, tibiae, and fibulae generated using the two‐phase rigid method, with a red square pinpointing an osteolytic bone lesion. Other osteolytic bone lesions are evident as bright spots in the patients' femurs. Figure 4 A2 and A4 are generated using the nonrigid reference method but the patients are the same as in Figure 4 a1 and a3. The lesions highlighted by red squares are less evident.

Figure 4 B1–B4 display an axial slice of the lesion region before the onset of the disease. Figure 4 b2 and b4 has been registered nonrigidly, and thus have become warped, as careful observation reveals.

Figure 4 c1–c4 show the same axial slice after the onset of MM. In the post‐MM images, bone marrow infiltrate is observable as a gray mass in the post‐MM images Figure 4 c1 and c2. Figure 4 d1–d4 shows the results of temporal subtraction. The osteolytic lesions are visible as high‐intensity voxels. The temporal change is more visible in Figure 4d1 and d3, using the rigid method than in Figure 4d2 and d4, using the nonrigid method.

In Figure 4 d1 and d2 adjacent to the osteolytic bone lesion is an area of heightened density due to bone marrow infiltrate, visible in black.

Figure 5 visually compares the rigid and nonrigid registrations. In Column a are slices of post‐MM images that show a loss of bone compared to rigidly registered pre‐MM images of Column b. In Column c are the same pre‐MM images and slices nonrigidly registered. Careful inspection of the blue squared area and comparing it to the same area in Column b shows that the nonrigid registration has warped lesion‐like dents into the inner surfaces of the pre‐MM bones. Images of Column d further visualize the results of this unwanted warping effect. In Column d, nonrigidly registered pre‐MM images have been subtracted from rigidly registered pre‐MM images, highlighting differences between the two methods.

## 4 DISCUSSION

The proposed automated pipeline exhibits promising results for tracking temporal changes in bone structures, particularly focusing on femurs, tibiae, and fibulae. We needed a highly reliable and consistent bone segmentation method to design a fully automated pipeline. The obvious choice for this task was the utilization of a CNN. The segmentation accuracy of the CNN was notably high, demonstrated by a mean DSC of 0.928 across all bone types and all test data. Moreover, considering the images' voxel spacing, the mean surface distances fell within acceptable ranges, thus signifying precise segmentation.

In assessing temporal alterations in bone structures, utmost precision in registrations is essential, given that registration errors manifest as temporal changes in bone composition within the difference image between two time points. For this reason, we developed a robust rigid registration pipeline. The bone‐wise rigid two‐phase registration process displayed commendable accuracy. DSC values exceeding 0.93 and MSDs averaging around 0.3 to 0.4 mm for femurs and tibiae indicated successful alignment between pre‐ and post‐MM images. We also validated the two‐phase registration strategy by registering the bones without the second phase and with a much simpler baseline registration method. The second phase, measured with DSC and MSD, improved the registrations of right fibulae from 0.90 to 0.93 DSC and from 0.42 mm MSD to 0.23 mm. The notably lower registration accuracies (mean 0.85) of the baseline method served to contrast our rather complex strategy. Measured by MSE, the baseline registration performed the worst and the second phase of registration improved the registration of tibiae and fibulae. The registration results were further validated through visual confirmation by clinically experienced researchers using contour overlays of registered segmentation masks and difference images generated by the temporal subtraction method. However, the registration pipeline was adjusted for the test data set due to limitations of available data and it has not been tested with other data. It should be noted that registration accuracy, measured in DSC and MSD, is limited by the performance of the CNN. Small deviations in the segmentations between two time points will render metrically perfect registration impossible as the binary masks that are used in the calculation of the metric are slightly different.

Our decision to forego the widely used registration accuracy metric, target registration error (TRE),^[^ ^34^ ^]^ stemmed from our specific research focus. Unlike TRE, which measures the absolute registration error, our primary interest, in terms of registration accuracy, lies in determining the comparative performance among different methods. Instead of focusing on absolute registration errors, our goal was to assess and compare the relative performance of various methods. By employing metrics like the DSC, MSD, and MSE, we aimed to evaluate how well different techniques aligned or segmented datasets concerning each other, allowing us to discern the superior method without emphasizing absolute registration errors. This approach enabled us to determine the method that exhibited the most favorable performance relative to others in our study context. In addition, by not using TRE, we avoid the difficulties in accurately locating corresponding landmarks and the associated target localization error. Moreover, the objects of our interest, osteolytic bone lesions, cannot be used as targets as they are present only in the post‐MM pictures.

The inclusion of MSE as an evaluation metric for registration accuracy, in addition to DSC and MSD, stems from its ability to capture nuances beyond the scope of DSC and MSD. Unlike DSC and MSD, which reflect the alignment of binary masks to assess spatial overlap and surface differences, MSE quantifies the differences in voxel intensities between registered images. This additional metric allows us to consider subtle variations in voxel intensity, providing a more comprehensive understanding of registration accuracy.

In scientific literature regarding bone‐related temporal subtraction, authors often report^[^ ^6^, ^7^, ^9^ ^]^ the omission of the segmentation step and the use of nonrigid registration. The inherent problem with the nonrigid approach is the possible loss of temporal information due to localized deformations as the algorithm maximizes similarity between two images. While these deformations aid in maximizing the alignment between images, they simultaneously may hinder the accurate extraction or analysis of temporal changes, potentially impacting the overall reliability and interpretability of the results obtained through nonrigid registration techniques.

We contrasted our method by comparing it to a nonrigid registration approach, but while in terms of performance evaluating parameters the nonrigid method performs better, the problems induced by the deformations of the nonrigid algorithm are clear upon inspecting the actual registered images and the differences images generated from those. The nonrigid method caused warping in the moving images of the registration process, which resulted in diminished intensity in the difference images. This increases the threshold of disease detection. While we cannot make definitive statements about the clinical impact of the effect of the nonrigid warping on disease detection from the limited dataset we have, we can say for certain that this effect can be entirely eliminated using rigid methods. In our assessment, the most accurate temporal subtraction, for rigid objects, is produced by piecewise segmentation and registration.

Up to 90% of MM patients develop osteolytic lesions during the course of the disease.^[^ ^35^ ^]^ These lesions occur predominantly in the axial skeleton and the most common sites include the vertebrae (66% of patients), ribs (45%), skull (40%), and pelvis (30%), whereas the involvement of the distal bones is more unusual.^[^ ^36^ ^]^ Our study focused on the long bones of the lower limb because the tubular bone structure allows accurate detection of myeloma lesions, not only in cortical and trabecular bone but also in the bone marrow. Myeloma infiltrates in the bone marrow can be detected by CT if they are located in the medullary spaces of long bones where they separate from the fatty bone marrow.^[^ ^37^ ^]^ The proposed automated pipeline tool could also be extended to cover axial bones and upper extremity long bones to reveal osteolytic bone disease and temporal alterations in repeated whole‐body CT scans of MM patients. However, CT‐based assessment of the extent of intramedullary bone disease in axial trabecular bones, such as vertebral bodies is more challenging due to the trabeculae themselves as well as degenerative changes, general osteoporosis, and benign bone lesions, such as vertebral hemangiomas.^[^ ^38^, ^39^ ^]^

Almost all patients with MM will eventually relapse and the choice of a treatment regimen at relapse is affected by many factors, including the timing of relapse, aggressiveness of relapse, response to prior therapy, and performance status.^[^ ^40^ ^]^ Therefore, forecasting the possible relapse as early as possible is important to foresee a therapy. The difference images, created to highlight temporal changes, effectively showcased osteolytic bone lesions and bone marrow infiltrate within the bone marrow. The demonstrated pipeline successfully tracked changes, providing a valuable tool for monitoring disease progression and showing potential for defining relapsed and non‐relapsed disease in patient follow‐up and treatment. Finally, the automatic detection of temporal changes in bone structures could potentially reduce the variability of image interpretation between readers.

Other metastatic tumor infiltrates in bone marrow, such as prostate and breast cancer metastases, can cause bone responses that vary with the type of malignancy. This results in either lytic, sclerotic, or mixed lytic‐sclerotic bone lesions, which can change both in size and morphology due to treatment effects. This automated tracking of temporal changes in bone lesion characteristics could also enable objective measurements of these different metastatic bone lesions and improve the accuracy of patient treatment monitoring.

However, certain limitations need acknowledgment. The relatively small dataset constrained the pipeline's performance evaluation, impacting the results' generalizability. Future work should involve a more extensive dataset and testing the pipeline on longitudinal post‐MM diagnosis data. Also, our study is limited by the lack of absolute ground truth data, for example, perfect ground truth registrations and radiologist's annotation of every single osteolytic bone lesion. Our pipeline is an effective way of generating a visualization aid for the human eye, but it does not remove the human eye and human error from the equation. Training accurate deep learning‐based detection methods would require a substantially larger dataset. Additionally, the versatility of our methodology allows for potential extension to encompass additional bone structures relevant to MM, such as the axial skeleton.

## 5 CONCLUSION

In this study, we introduced a comprehensive and automated pipeline for accurately tracking temporal changes in bone structures, with a specific focus on femurs, tibiae, and fibulae, in patients affected by MM. The pipeline encompasses a CNN for precise 3D bone segmentation, followed by a meticulously designed bone‐wise rigid registration process. Difference images were generated by temporal subtraction to visualize temporal alterations, effectively highlighting areas of interest, such as osteolytic bone lesions.

The CNN segmentation exhibited a sufficient level of accuracy, as demonstrated by high DSCs and MSDs. Furthermore, our bone‐wise registration pipeline showcased its efficacy in accurately aligning bone structures, enabling a reliable assessment of temporal changes.

The methodology we presented not only lays a solid foundation for tracking temporal bone alterations in MM patients but also offers flexibility for extension to include additional bone structures, such as the spinal cord. These advancements may contribute to a more comprehensive understanding of disease progression and could empower clinicians to make informed decisions regarding patient treatment strategies.

This research represents a stride toward automated and precise temporal tracking of bone alterations, that could ultimately contribute to improved patient care and outcomes in the context of MM.

## AUTHOR CONTRIBUTIONS

**Arttu Ruohola**: The main image analysis and drafted the manuscript. **Ville Haapamäki**: Drafted the manuscript, interpreted the data, annotated images, collected data and designed the study. **Eero Salli**: Reviewed and edited the manuscript, prepared study data, and designed the study and methodology. **Tuomas Kaseva**: Auxiliary image analysis to confirm the results and revised the manuscript and wrote neural network segmentation scripts. **Marko Kangasniemi**: Conceptualization, methodology, project administration, manuscript review, and editing. **Sauli Savolainen**: Conceptualization, methodology, project administration, manuscript review, and editing.

## CONFLICT OF INTEREST STATEMENT

The authors have no conflict of interest to disclose.

## Appendix

The flowcharts of Figures A1 and A2 visualize of the utilized segmentation and registration processes, respectively.
